# Development of membrane protein‐based vaccine against lumpy skin disease virus (LSDV) using immunoinformatic tools

**DOI:** 10.1002/vms3.1438

**Published:** 2024-03-31

**Authors:** Md. Salauddin, Mohammad Enamul Hoque Kayesh, Md. Suruj Ahammed, Sukumar Saha, Md. Golzar Hossain

**Affiliations:** ^1^ Department of Microbiology and Public Health Khulna Agricultural University Khulna Bangladesh; ^2^ Department of Microbiology and Public Health Patuakhali Science and Technology University Barishal Bangladesh; ^3^ Department of Chemistry Bangladesh University of Engineering and Technology Dhaka Bangladesh; ^4^ Department of Microbiology and Hygiene Bangladesh Agricultural University Mymensingh Bangladesh

**Keywords:** dynamic simulations, lumpy skin disease virus, membrane glycoprotein B and T cells, molecular docking, PET29a (+) vector, vaccine

## Abstract

**Introduction:**

Lumpy skin disease, an economically significant bovine illness, is now found in previously unheard‐of geographic regions. Vaccination is one of the most important ways to stop its further spread.

**Aim:**

Therefore, in this study, we applied advanced immunoinformatics approaches to design and develop an effective lumpy skin disease virus (LSDV) vaccine.

**Methods:**

The membrane glycoprotein was selected for prediction of the different B‐ and T‐cell epitopes by using the immune epitope database. The selected B‐ and T‐cell epitopes were combined with the appropriate linkers and adjuvant resulted in a vaccine chimera construct. Bioinformatics tools were used to predict, refine and validate the 2D, 3D structures and for molecular docking with toll‐like receptor 4 using different servers. The constructed vaccine candidate was further processed on the basis of antigenicity, allergenicity, solubility, different physiochemical properties and molecular docking scores.

**Results:**

The in silico immune simulation induced significant response for immune cells. In silico cloning and codon optimization were performed to express the vaccine candidate in *Escherichia coli*. This study highlights a good signal for the design of a peptide‐based LSDV vaccine.

**Conclusion:**

Thus, the present findings may indicate that the engineered multi‐epitope vaccine is structurally stable and can induce a strong immune response, which should help in developing an effective vaccine towards controlling LSDV infection.

## Graphical abstract

Following the retrieval of the Membrane glycoprotein of LSDV (Gene Bank ID: QIN91553.1), it was subjected to analysis for B‐ and T‐cell epitopes and other parameters. Subsequently, the construct for the 3D structure of the vaccine chimera was generated. Molecular docking with TLR4, covariance map analysis, in silico immune simulation and cloning were performed utilizing the pET29a (+) vector.

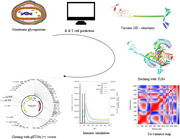

## 1 INTRODUCTION

Lumpy skin disease (LSD) is an economically important vector‐borne transboundary disease of ruminants caused by lumpy skin disease virus (LSDV) (Kayesh et al., 2020). LSDV is a double‐stranded DNA virus under the family Poxviridae and the genus *Capripoxvirus* (Sprygin et al., 2019; Tulman et al., 2001). LSDV contains a large genome of about 151 kb that codes for 156 putative genes and with 2 identical inverted terminal repeat regions of about 2.4 kb at both ends of the central coding region (Tulman et al., 2001). The genus *Capripoxvirus* contains three very closely related animal viruses such as sheeppox virus (SPPV), goatpox virus (GTPV) and LSDV, and serologically, these viruses cannot be separated (Babiuk et al., 2008; Badhy et al., 2021). LSD is mechanically transmitted by blood‐feeding mosquitos, biting flies and ticks (Chihota et al., 2001; Sanz‐Bernardo et al., 2021; Sohier et al., 2019; Tuppurainen et al., 2013). LSDV has a narrow host range, infecting cattle (*Bos indicus* and *Bos taurus*) and buffaloes (*Bubalus bubalis*) (Kar et al., 2022). The *B. taurus* cattle is more susceptible to LSDV compared to the *B. indicus* cattle (Gupta et al., 2020). LSDV causes significant economic losses in cattle and buffalo industry globally, which causes enlisting of LSD as a World Organization for Animal Health (WOAH/OIE)‐notifiable disease. Previously, LSD was endemic only in African countries; however, recently, it has spread to many Asian cattle‐rearing countries (Khan et al., 2021).

Until now, there is no antiviral treatment for LSDV infection (Uddin et al., 2022). Although strict quarantine measures and vector control are essential for preventing its spreading, however, vaccination remains the mainstay of preventing the spread of the infection in endemic areas as well as in newly affected regions (Tuppurainen et al., 2021). Vaccination is crucial for maintaining the good health of animals and the economic sustainability of cattle and buffalo farming against LSD threats (Tuppurainen et al., 2021). For controlling LSDV infection, homologous vaccines consisting of live attenuated LSDV can be used (Sprygin et al., 2020). Due to cross‐protection within the *Capripoxvirus* genus, heterologous vaccines consisting of live attenuated SPPV or GTPV can also be used for controlling LSDV infection (Tuppurainen et al., 2021). However, the vaccine efficacy is not consistent rather varies with quality, efficacy, safety, side effects and even price (Tuppurainen et al., 2021). Moreover, there are the issues of improper characterization of circulating wild strains as well as viral vaccine strains in terms of host specificity, vaccination failures and safety concerns with capripox vaccinations (Sumana et al., 2020). Moreover, the administration of heterologous vaccinations in Turkey was not able to stop LSD outbreaks, indicating that the success of the vaccine is not adequate, necessitating the development of new vaccine candidate for control and prevention of LSD.

Immunoinformatics and computational vaccinology are useful in providing insight into the host‐pathogen interactions thus playing an important role in vaccine development. Immunoinformatics and computational vaccinology become an invaluable tool for more rapid and precise vaccine design (Ishack & Lipner, 2021). The design of a multi‐epitope vaccine targeting viral structural and non‐structural proteins through immunoinformatics tools appears promising (Behmard et al., 2022; Fadaka et al., 2021; Huang et al., 2022). However, there is very limited information on immunoinformatics approaches to develop LSD vaccine. Therefore, in this study by applying immunoinformatics approaches, we have designed possible vaccine candidates targeting LSDV. However, the designed vaccine candidates are required to be further experimentally investigated for the efficacy as potential vaccines against LSDV infection.

## 2 MATERIALS AND METHODS

### 2.1 Retrieval of protein sequences and structural analysis

The gene sequence of intracellular enveloped virus (IEV) and extracellular enveloped virions (EEV) membrane glycoprotein of LSDV was obtained from National Centre for Biotechnology Information Database (https://www.ncbi.nlm.nih.gov/) (GenBank accession number QIN91553.1). The physiochemical properties of the selected protein were analysed by using ExPASy ProtParam tool (Gasteiger et al., 2005). The secondary structure analysis of protein was conducted by position‐specific iterated prediction (PSIPRED) and Garnier–Osguthorpe–Robson (GOR IV) online tools (Buchan et al., 2013; Garnier et al., 1996). Determination of the antigenic protein was done by Vaxijen 2.0 (Doytchinova & Flower, 2008), and the protein showed that the most antigenicity was selected for further analysis. Allergenicity and toxicity were detected by using AllerTOP v2.0 and ToxinPred, respectively (Dimitrov et al., 2013; Gupta et al., 2013). Secondary structure prediction of the selected M protein was analysed by PsiPred tool to investigate the peptidyl structure. For transmembrane (TM) helix prediction, the TMHMM v2.0 (http://www.cbs.dtu.dk/services/TMHMM/) based on the hidden Markov model (HMM) was utilized (Krogh et al., 2001). Finally, structural proteins that showed most antigenicity and non‐allergenicity with less TM helices were selected for further investigation.

### 2.2 Prediction of MHC I epitopes

Prediction of major histocompatibility complex (MHC) class I molecules (MHC I) binding of conserved epitopes was accomplished by immune epitope database (IEDB) MHC I Binding Predictions Tool (http://tools.iedb.org/mhci) (Buus et al., 2003). Sequence submission was done in FASTA format, and the prediction method was set as artificial neural networks (ANN). Host species was set as cow. All the 7‐alleles (BoLA‐AW10, BoLA‐D18.4, BoLA‐HD6, BoLA‐JSP.1, BoLA‐T2a, BoLA‐T2b and BoLA‐T2c) were selected with a length of 9. Output format was selected as extensible hypertext markup language (XHTML) table, and all the other options and parameters were set as default.

### 2.3 Prediction of MHC II epitopes

The IEDB MHC II server (http://tools.iedb.org/mhcii/) was used for the prediction of helper T lymphocyte epitopes (Andreatta et al., 2015; Zhang et al., 2008). FASTA format of sequence was used for submission, and the prediction method was set to NN‐align 2.3 (NetMHCII 2.3). The species/locus was chosen as human/HLA‐DR, HLA‐DQ and HLA‐DP, and all the alleles were chosen at default length parameters. Further, 15‐mer epitopes were retrieved and classified as per the percentile value. Output format was set as XHTML table, and the other parameters were set as default.

### 2.4 Prediction of linear B‐cell epitopes

The potential linear B‐cell epitopes were selected by BepiPred (http://tools.iedb.org/bcell/) from the IEDB research resource (Larsen et al., 2006). In addition to select the linear B‐cell epitopes, BepiPred also predicts continuous epitopes by combining two residue properties with the HMM.

### 2.5 Prediction of antigenicity, allergenicity and toxicity of B‐ and T‐cell epitopes

A vaccine candidate must have the antigenicity, which can be measured by freely accessible online tool VaxiJen v2.0 (http://www.ddg‐pharmfac.net/Vaxijen/VaxiJen/VaxiJen.html). For the vaccine candidate construction, the antigenic evaluation of the selected epitopes was performed with a threshold value of ≥0.4 (Doytchinova & Flower, 2007). Allergen identification is also an important factor for vaccine development. AllerTOP v.2.0 server was used for measuring the allergenic properties of the vaccine candidates. AllerTOP v2.0 is an online tool (http://www.ddg‐pharmfac.net/AllerTOP) that utilizes the k nearest neighbours, auto‐ and cross‐covariance transformation and amino acid E‐descriptors machine learning techniques for the classification of allergens by exploring the physiochemical properties of proteins. The accuracy of this approach was stated as 85.3% at fivefold cross‐validation (Dimitrov et al., 2014; Magnan et al., 2010). The non‐allergenic protein sequences were selected for further analysis. Finally, all the epitopes were checked for toxicity using the ToxinPred (https://webs.iiitd.edu.in/raghava/toxinpred/multi_submit.php) (Gupta et al., 2015), and non‐toxic epitopes were chosen. The whole vaccine construct was also investigated for these characteristics.

### 2.6 Vaccine construction

Vaccine construction was done by obtaining adjuvant sequence from NCBI, and all potential epitopes were exploited to develop the multi‐epitope vaccine construct. To enhance the immunogenicity of the vaccine candidate, a toll‐like receptor 4 (TLR4)‐inducing 50S ribosomal protein L7/L12 (UniProt ID: P9WHE3) was used as an adjuvant, which was linked to the N‐terminus of the vaccine candidate. Four different linkers such as EAAAK, AAY, GPGPG and KK were used. 6× His tag was added at the C‐terminal to carry out the expression and binding of histidine protein.

### 2.7 Assemblage of multi‐epitopic vaccine candidate sequence

To obtain the complete sequence of the candidate vaccine adjuvant sequence, linker sequences of EAAAK, AAY, GPGPG and KK linkers, and the sequences of MHC I, MHC II and B‐cell epitopes were manually merged (Majid & Andleeb, 2019; Sami et al., 2021). The sequence of merged final vaccine construct is shown in Tables 1–3.

**TABLE 1:** Prediction of major histocompatibility complex (MHC) I epitopes.

MHC I			
Epitopes	Antigenicity	Allergenicity	Toxicity
GLVKKKNNI	1.8445	Non allergen	Non toxic
LTYGRQFWY	0.7367	Non allergen	Non toxic
AIFMLVSTI	0.4443	Non allergen	Non toxic
YVSYIICVK	1.0688	Non allergen	Non toxic
VSYIICVKR	0.9729	Non allergen	Non toxic
LQLSLYGGV	0.6519	Non allergen	Non toxic
GQFKNVSCN	0.6314	Non allergen	Non toxic

### 2.8 Physiochemical properties and solubility prediction

Physicochemical properties of the vaccine construct like theoretical isoelectric point (pI), composition of amino acid, molecular weight (MW), in vitro and in vivo half‐life, instability and aliphatic index and grand average of hydropathicity (GRAVY) were predicted by using Expasy ProtParam (https://web.expasy.org/protparam/) (Gasteiger et al., 2005). The solubility of multi‐epitope vaccine was estimated using the protein–sol (http://protein‐sol.manchester.ac.uk). The scaled solubility value (QuerySol) was considered the predicted solubility. The population average for the experimental dataset (PopAvrSol) was set at 0.45, and any scaled solubility value greater than 0.45 was considered for a higher solubility than the average soluble *Escherichia coli* protein from the experimental solubility dataset (Hebditch et al., 2017; Sippl, 1993; Wiederstein & Sippl, 2007). Similarly, protein with a lower scaled solubility value was considered to be less soluble.

### 2.9 Antigenicity, allergenicity and toxicity prediction of vaccine construct

The antigenicity of the multi‐epitope vaccine construct was analysed by Vaxigen 2.0 (Doytchinova & Flower, 2007), and the allergenicity of multi‐epitope vaccine construct was predicted using AllerTOP (Dimitrov et al., 2014; Magnan et al., 2010). Furthermore, toxicity analysis of the construct was done using ToxinPred (Gupta et al., 2015).

### 2.10 Secondary structure prediction

The secondary structure of the multi‐epitope vaccine construct was conducted using the GOR IV online tool (https://npsa‐prabi.ibcp.fr/cgi‐bin/npsa_automat.pl?page=npsa_gor4.html) with a mean accuracy of 64.4% (Buchan et al., 2013; Garnier et al., 1996), and PSIPRED was based on outputs from PSIBLAST (https://bioinf.cs.ucl.ac.uk/psipred/). PSIPRED is an online server of efficient secondary structure prediction of the TM topology, TM helix, fold and domain recognition and so on (Buchan & Jones, 2019; Garnier et al., 1978).

### 2.11 Tertiary structure prediction

The tertiary or three‐dimensional (3D) modelling of the multi‐epitope vaccine was done using transform restrained Rosetta (trRosetta) server (https://yanglab.nankai.edu.cn/trRosetta/). trRosetta is an algorithm for fast and accurate protein structure prediction, building the protein structure based on direct energy minimizations with a restrained Rosetta. The restraints include inter‐residue distance and orientation distributions, predicted by a deep neural network architecture. To improve the accuracy, further homologous templates are included in the network prediction. In benchmark tests on CASP13 and CAMEO‐derived sets, trRosetta predicts better than other previously described methods (Du et al., 2021; Su et al., 2021; Yang et al., 2020).

### 2.12 Refinement of the tertiary structure

To further improve the 3D model of the multi‐epitope vaccine construct, the GalaxyRefine web server (http://galaxy.seoklab.org/cgi‐bin/submit.cgi?type = REFINE) has been utilized using CASP10 refinement techniques. This tool can improve both global and local structure quality (Heo et al., 2013).

### 2.13 Validation of tertiary structure

Tertiary structure validation is a difficult step of the model construction approach because it finds possible errors in the predicted 3D models (Khatoon et al., 2017). ProSA‐web server (https://prosa.services.came.sbg.ac.at/prosa.php) was initially used for 3D structure validation of protein, which estimates a total quality score of the exact input structure shown as *Z*‐score. *Z*‐scores when remain outside the range of the properties for native proteins, suggesting that the structure likely contains errors (Wiederstein & Sippl, 2007). A Ramachandran plot was retrieved via RAMPAGE web‐server (http://mordred.bioc.cam.ac.uk/~rapper/rampage.php) and describes the quality of the modelled structure by depicting the percentage of residues in disallowed and allowed regions (Lovell et al., 2003). The model was further validated by PROCHECK (Laskowski et al., 1993).

### 2.14 Prediction of discontinuous B‐cell epitopes

More than 90% of B‐cell epitopes are known to be discontinuous in nature. ElliPro, a web‐tool (http://tools.iedb.org/ellipro/) was used to predict the discontinuous (conformational) B‐cell epitopes in the validated 3D structure. ElliPro employs three algorithms based on their protrusion index (PI) values to estimate the protein structure as an ellipsoid and measures the residue PI, and adjacent cluster residues. ElliPro offers a score for each predicted epitope defined as a PI value averaged over epitope residues. The ellipsoid with a PI value of 0.9 is deemed as 90% protein residues are included, whereas the remaining 10% residues are outside of ellipsoids. The PI value for each epitope residue was calculated on the basis of the centre of residue mass residing outside the largest ellipsoid possible. Compared to other structure‐based methods that are used to predict epitopes, ElliPro remains as the top and provides an AUC value of 0.732, when the most significant prediction was considered for any protein.

### 2.15 Disulfide engineering

Disulfide bonds are covalent interactions offering considerable stability and strengthen the geometric conformation of proteins. The disulfide engineering of the vaccine construct was conducted with the online DbD2 server (http://cptweb.cpt.wayne.edu/DbD2/) to confer stability enhancement of the protein. This web tool predicts the pairs of residues able to form a disulfide bond, if amino acid residue is mutated to cysteine (Craig & Dombkowski, 2013).

### 2.16 Molecular docking of the final vaccine with immune receptor

Cluspro2.0 online docking server (https://cluspro.bu.edu/) was used to carry out the molecular docking analysis of TLR4 ligand binding domain (PDB ID: 4G8A; https://www.rcsb.org/structure/4G8A) and the vaccine construct (Kozakov et al., 2017). This tool was considered one of the best docking servers due to its number of advanced option of searches according to requirement.

### 2.17 Molecular dynamics simulation

The iMODS (http://imods.Chaconlab.org/) simulation server can explore the collective motions of proteins and nucleic acids using normal modes in internal coordinates (Van Aalten et al., 1997). The iMODS web‐server was used for the molecular dynamics simulation to define and calculate the protein flexibility (Lopéz‐Blanco et al., 2011). Compared to the other processes of molecular dynamics simulations, it is quicker and cost‐efficient (Tama & Brooks, 2006). This web server is used for the prediction of the eigenvalues, deformability, B‐factors and covariance. The eigenvalues are used for the motif stiffness assessment, whereas the deformity of the main chain is predicted from the biological targets’ efficacy measurement (Tama & Brooks, 2006).

### 2.18 Codon optimization and in silico cloning

To express the chimeric protein in an expression system, the sequence of the vaccine construct was reverse translated by the backtranseq program of EMBOSS 6.0.1 (https://www.ebi.ac.uk/Tools/emboss/), followed by codon optimization using Java Codon Adaption tool (JCat) (http://www.jcat.de/), an online web‐based program (Grote et al., 2005). Java Codon Adaptation Tool server was used in the codon system of *Drosophila melanogaster* to obtain the codon adaptation index (CAI) values and GC contents to determine the levels of protein expression. The best CAI value is 1.0, whereas >0.8 is considered a good score, and the GC content ranges between 30% and 70% (Morla et al., 2016), and beyond this range may effect on translation and transcriptional efficiencies (Ali et al., 2017). The optimized gene sequence of multi‐epitope vaccine was cloned in *E. coli* plasmid vector pET 29a (+), PpuMI and TatI restriction sites were added to the N‐ and C‐terminals of the sequence, respectively. Finally, to confirm the expression of the vaccine, the final vaccine construct (with restriction sites) was inserted into the plasmid vector pET 29a (+) using the SnapGene software (https://www.snapgene.com/free‐trial/).

### 2.19 Immune simulation

To determine the immune response of the vaccine construct, an immune simulation was performed by a web‐based simulation server, C‐ImmSim (http://kraken.iac.rm.cnr.it/C‐IMMSIM/). This server uses a position‐specific scoring matrix and machine learning for the prediction of both cellular and humoral immune response to the vaccine construct in mammals (Abraham Peele et al., 2021). Three injections of the candidate vaccine were administered at different intervals of 4 weeks. All simulation parameters such as random seed, number of antigens to inject and vaccine proteins (no LPS) were kept at their default settings with time periods set at 1, 84 and 168. The simulation volume and simulation steps were set at 50 and 1000, respectively.

## 3 RESULTS

### 3.1 Retrieval of protein sequences and structural analysis

**FIGURE 1:**
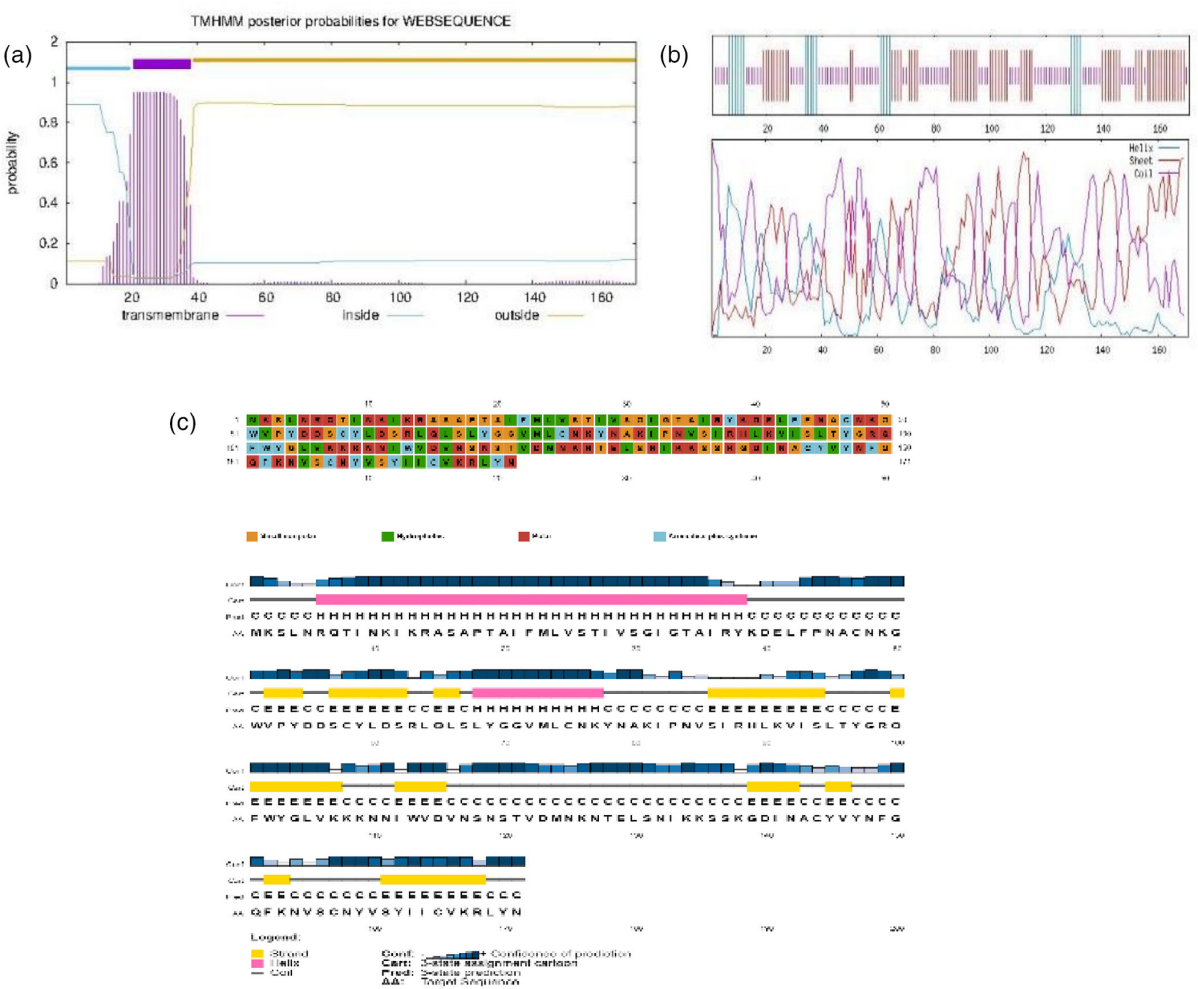
Results of retrieved sequence (a) TMHMM server, (b) results from Garnier–Osguthorpe–Robson (GOR IV) server and (c) 2D structure predicted by position‐specific iterated prediction (PSIPRED) online server.

The retrieved sequence was antigenic (0.5962; which indicates probable antigen), non‐allergen and non‐toxic. The expected half‐life was 30 h in mammalian reticulocytes (in vitro) >20 h in yeast (in vivo) and over 10 h in *E. coli* (in vivo) as estimated by ExPASy‐ProtParam. The GRAVY score was −0.226, whereas the protein instability index (II) was 35.84, indicating a stable protein. Moreover, the vaccine protein has 171 amino acids, and its MW was 19.51165 kDa. The theoretical pI was predicted to be 9.66. The chemical formula of the vaccine construct was C_875_H_1383_N_237_O_248_S_10_, composed of 2753 atoms and 89.42 aliphatic index. For TM helix prediction, the server showed a number of predicted TMHs: 1. possible N‐term signal sequence inside 1–20, TMhelix 21–38 and outside 39–171 (Figure 1a). GOR IV online server revealed that the alpha helix (Hh): 19 is 11.11%, extended strand (Ee): 66 is 38.60%, random coil (Cc): 86 is 50.29% (Figure 1b) and secondary structure predicted by PSIPRED online server (Figure 1c). This implies that the protein is suitable for vaccine design.

### 3.2 Prediction of MHC I and MCH II epitopes

MHC I epitopes were predicted using the IEDB MHC I web‐server fixed at the threshold value for epitope documentation. Among all the predicted MHC I epitopes, only seven epitopes were selected for vaccine construct based on their high scores binding affinity towards MHC I, antigenicity, non‐allergenicity and non‐toxicity, as shown in Table 1. MHC II binding peptides were predicted using the NN‐align 2.3 (NetMHCII 2.3), IEDB MHC II server. A total of six MHC II epitopes were selected for the final vaccine construct on the basis of binding affinity, antigenicity, non‐allergenicity and non‐toxicity (Table 2).

**TABLE 2:** Prediction of major histocompatibility complex (MHC) II epitopes.

MHC II			
Epitopes	Antigenicity	Allergenicity	Toxicity
HLKVISLTY	1.6335	Non allergen	Non toxic
VSCNYVSYI	1.1895	Non allergen	Non toxic
KRASAPTAI	0.5516	Non allergen	Non toxic
ISLTYGRQF	1.2993	Non allergen	Non toxic
LKVISLTYG	1.4699	Non allergen	Non toxic
LQLSLYGGV	0.6519	Non allergen	Non toxic

### 3.3 Prediction of linear B‐cell epitopes

The predicted B‐cell epitopes by BepiPred server with a cut‐off binding score >0.7, high antigenic, non‐allergenic and non‐toxic resulted in two B‐cell epitopes for final selection for vaccine construct as listed in Table 3.

**TABLE 3:** Prediction of linear B‐cell epitopes.

B cell epitopes			
Epitopes	Antigenicity	Allergenicity	Toxicity
KYNAKIPNVSI	0.9236	Non allergen	Non toxic
GLVKKKNNIWVDVNSNSTVDMNKNTELSNIKKSSKGDI	0.7491	Non allergen	Non toxic

### 3.4 Construction of multi‐epitope vaccine candidate sequence

**FIGURE 2:**
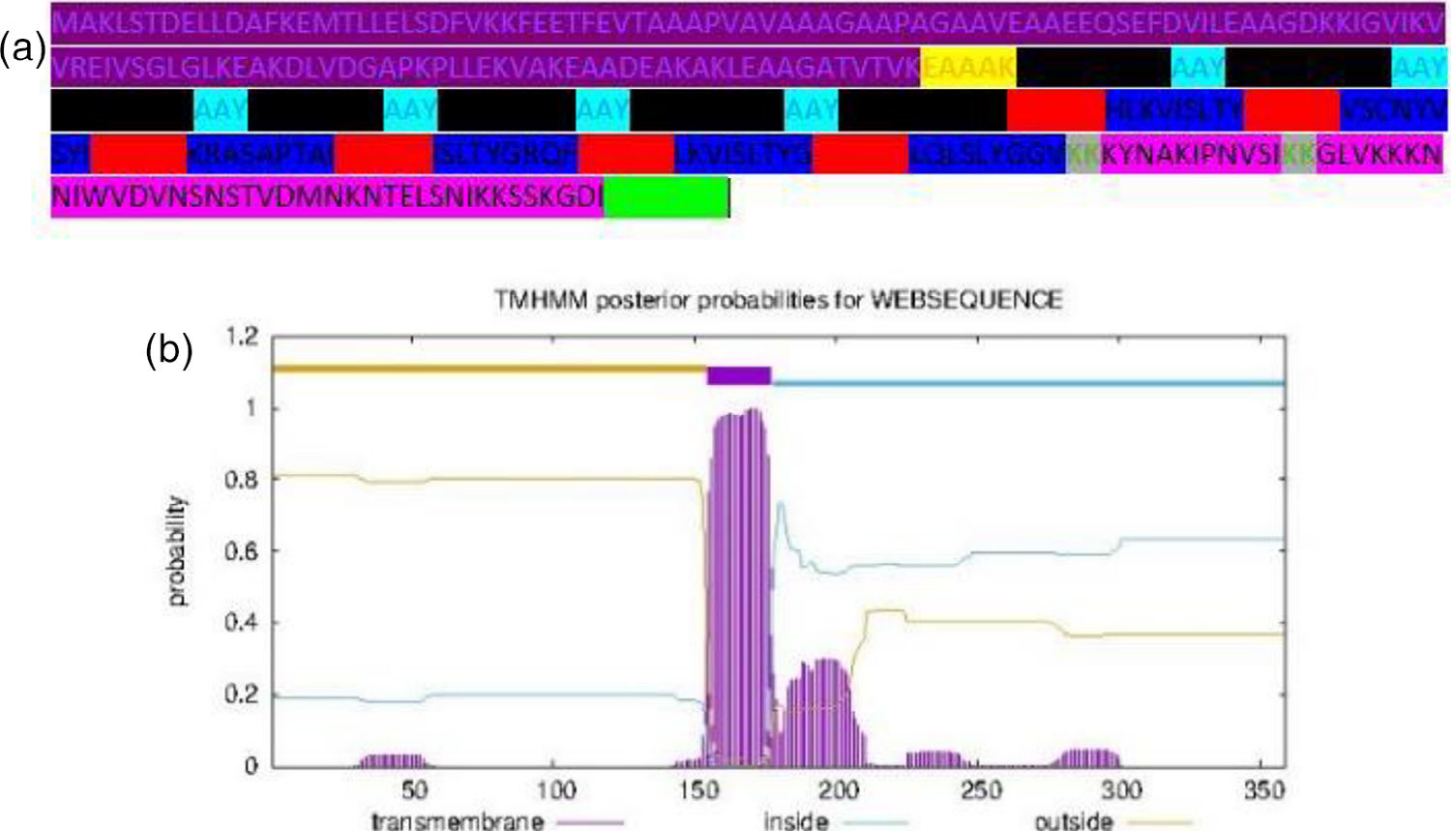
(a) Vaccine construct; purple: adjuvant, yellow: EAAAK, black: major histocompatibility complex (MHC) I, sky blue: AAY, red: GPGPG, blue: MHC II, grey: KK, pink: B cell epitope, green: 6× His tag, (b) TMHMM server output.

The finally selected six MHC I and seven MHC II‐binding epitopes and two B‐cell epitopes were used to design the multi‐epitope vaccine construct. An adjuvant 50S ribosomal protein L7/L12 (UniProt ID: P9WHE3) also added to the N‐terminal of the vaccine construct. EAAAK linker's link adjuvant to the epitopes, AAY linkers to link MHC I epitopes, GPGPG linker link to MHC II epitopes, KK linkers were used to link B‐cell, and at the C‐terminal, 6× His tag was added. The constructed vaccine sequence was again tested for antigenicity, non‐allergenicity, non‐toxicity, solubility and fulfilling all the criteria. The schematic presentation of the final multi‐epitope vaccine construct is shown in Figure 2a. TMHMM v2.0 server was used to check the TM helices of the structural proteins. The number of predicted TMHs was 1, possible N‐term signal sequence inside 178–359, TMhelix 155–177 and outside 1–154 (Figure 2b). Generally, proteins displaying >1 TM helices are not considered suitable vaccine targets, as these proteins are difficult to purify and ineffective in cloning and expression. Therefore, we excluded those proteins in vaccine design.

### 3.5 Physiochemical properties and solubility prediction

The chemical formula of the multi‐epitope vaccine construct is C_1721_H_2738_N_448_O_495_S_8_, containing 5410 atoms and 359 amino acids with an MW of 37.88186 kDa. The estimated pI of the vaccine construct was 9.29, which points out the alkaline nature of the vaccine candidate. The computed instability index was 20.92, aliphatic index of the construct was 93.54, and the GRAVY was 0.008, suggesting that the candidate vaccine is stable, polar in nature, and the candidate vaccine may be hydrophilic by nature and capable of interacting with the watery environment, as indicated by the GRAVY score of 0.008. The total number of negatively charged residues (Asp+ Glu) predicted to be 30, whereas the total number of positively charged residues (Arg+ Lys) was 43. The estimated half‐lives in mammalian reticulocytes (in vitro), yeast cells (in vivo) and *E. coli* (in vivo) were 30, >20 and >10 h, respectively.

### 3.6 Antigenicity, allergenicity and toxicity prediction of vaccine construct

**FIGURE 3:**
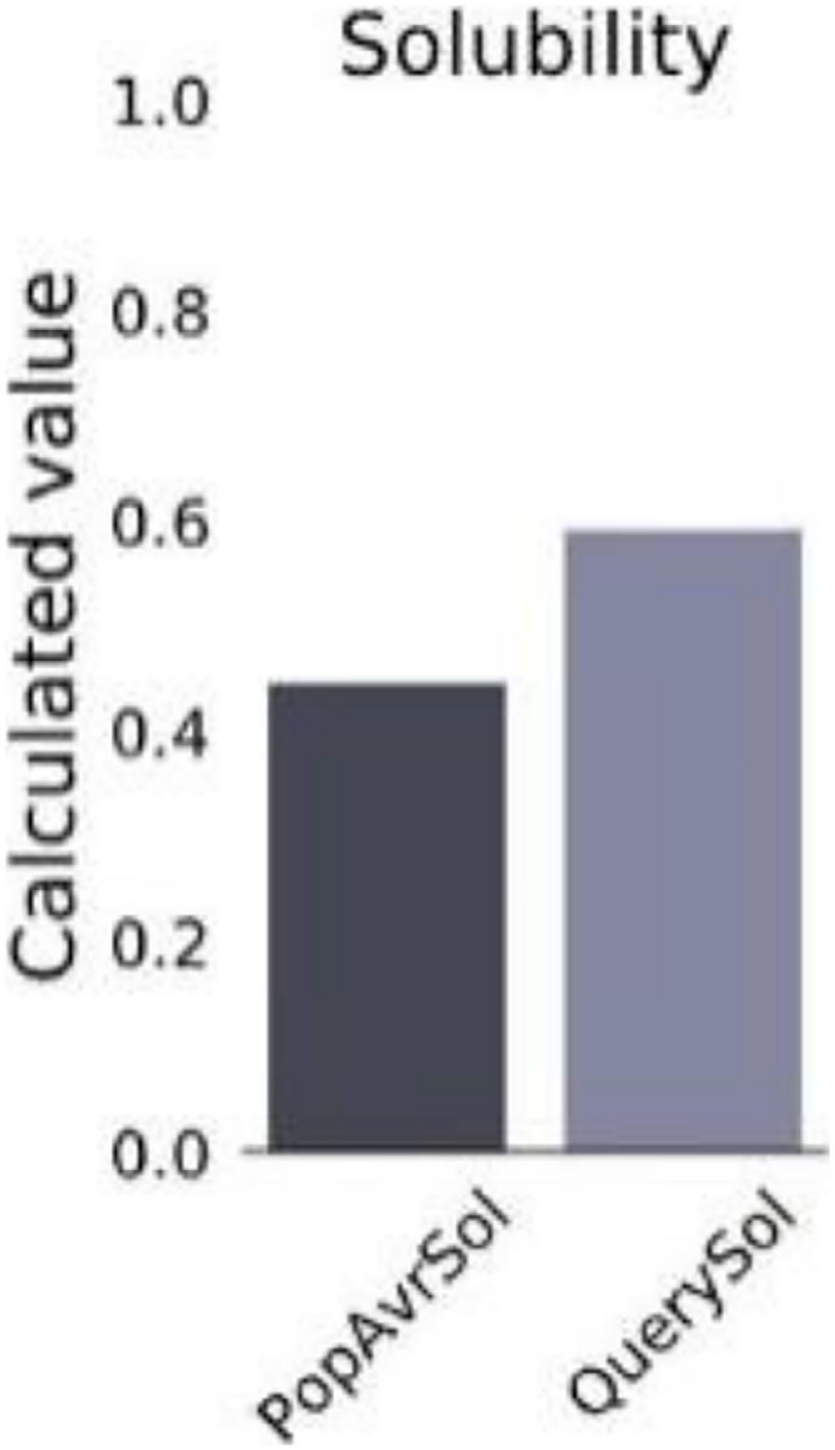
Solubility analysis of vaccine constructs using ProtSol with a score of 0.592 upon expression.

The antigenicity of the vaccine construct was analysed by VaxiJen v2.0 web tool, and the candidate antigen was found as a good antigen. In the VaxiJen v2.0 tool, the default threshold of 0.4 was chosen as the antigenicity criterion. The antigenicity score for multi‐epitope vaccine was 0.5878 as predicted by the VaxiJen v2.0 server. The vaccine construct was found non‐allergenic by AllerTOP v2.0. Protein Sol solubility revealed that the pI of the construct was 9.720, and the solubility was 0.592 (Figure 3). The solubility prediction using the SOLpro server when the protein structure is over‐expressed in *E. coli* illustrated that the vaccine construct is soluble with a probability of 0.912393. The results indicate that the constructed vaccine was highly antigenic. The vaccine sequence on ToxinPred server revealed the vaccine construct as non‐toxic.

### 3.7 Secondary structure prediction

**FIGURE 4:**
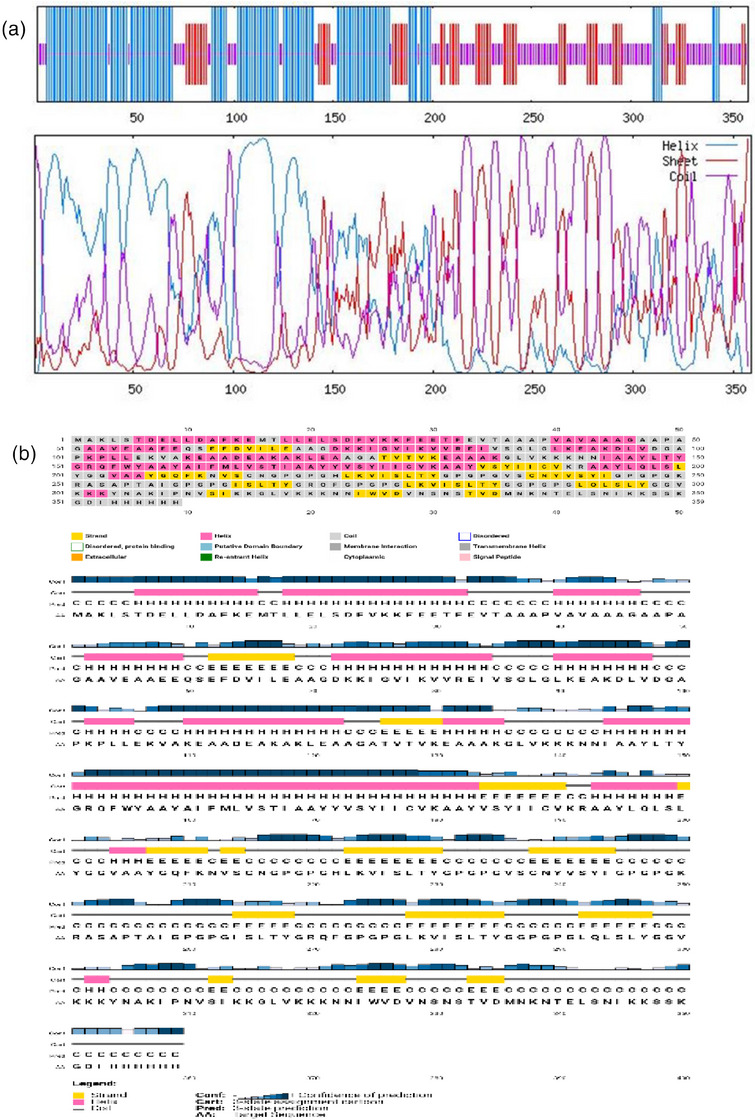
(a) Graphical output from Garnier–Osguthorpe–Robson (GOR IV) server, (b) secondary structure prediction of vaccine constructs using the position‐specific iterated prediction (PSIPRED) server having (42.90%) alpha helices, (20.33%) beta‐strands and (36.77%) random coils.

The candidate vaccine was estimated to have 42.90% α‐helix, 20.33% β‐strand and 36.77% random coil. Figure 4a shows the graphical descriptions of the GOR IV server and Figure 4b shows the performance of the secondary structure obtained from the PSIPRED server.

### 3.8 Tertiary structure prediction

**FIGURE 5:**
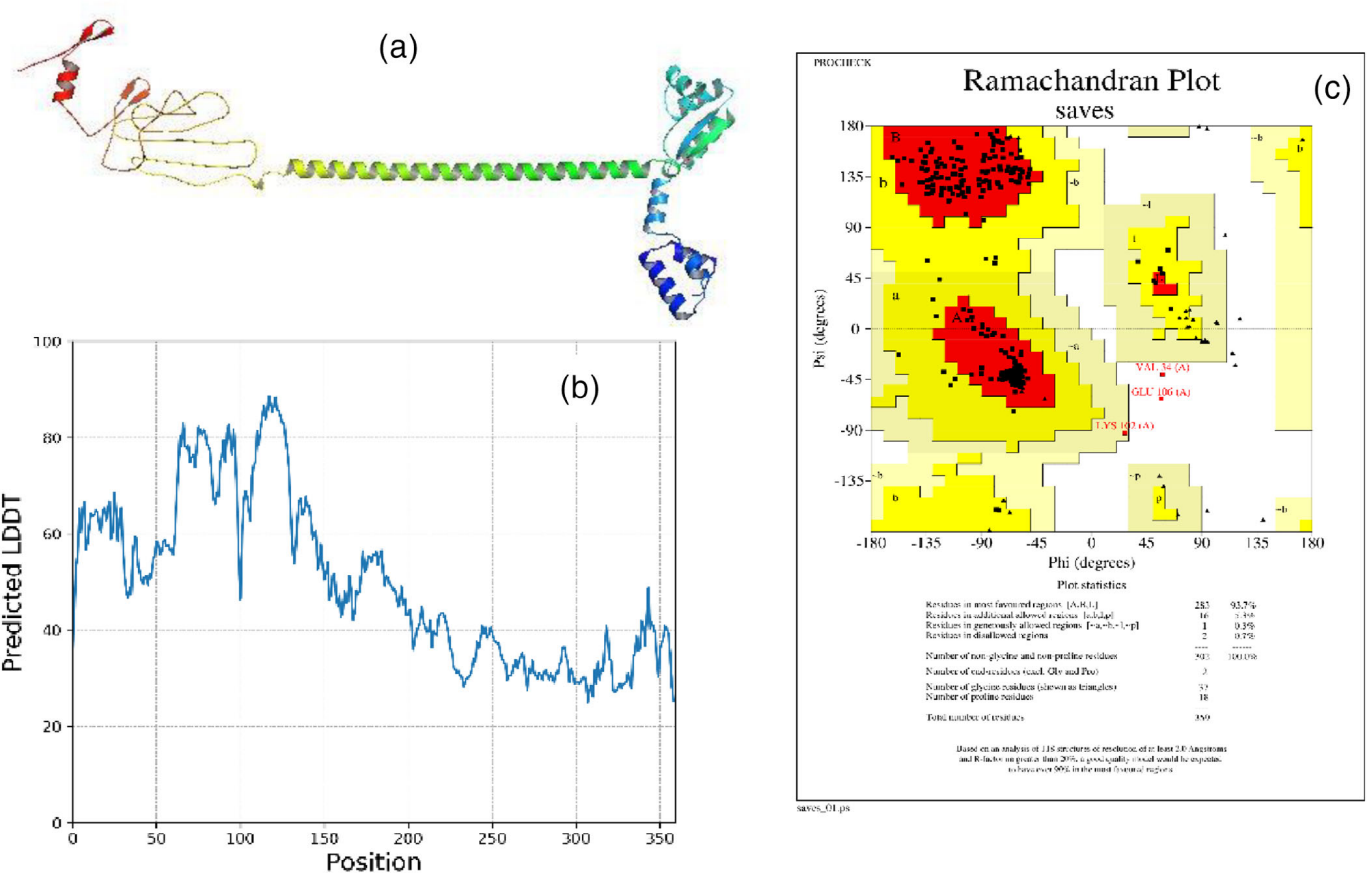
(a) Three‐dimensional (3D) structure of vaccine construct, (b) predicted pre‐residue local distance difference test (LDDT) for this model transmembrane (TM) 0.454, (C) Ramachandra plot before refinement.

The trRosetta server modelled five tertiary structures of the designed multi‐epitopic vaccine protein. The selected model had an estimated TM score of 0.454, and *Z*‐score was −3.05 by the ProSA‐web‐server (before refinement). The TM score indicates the structural similarity between two structures. A TM score of >0.5 indicates a model of correct topology, whereas a TM score of <0.17 suggests random similarity. 3D structure modelled by trRosetta was subjected to the PROCHECK server, where the Ramachandran plot was generated and the output revealed 93.7% residues that were present in the favoured region. The Ramachandran plot allows us to visualize energetically allowed and disallowed dihedral angles psi (*ψ*) and phi (*φ*) (Figure 5).

### 3.9 Refinement and validation of tertiary structure

**FIGURE 6:**
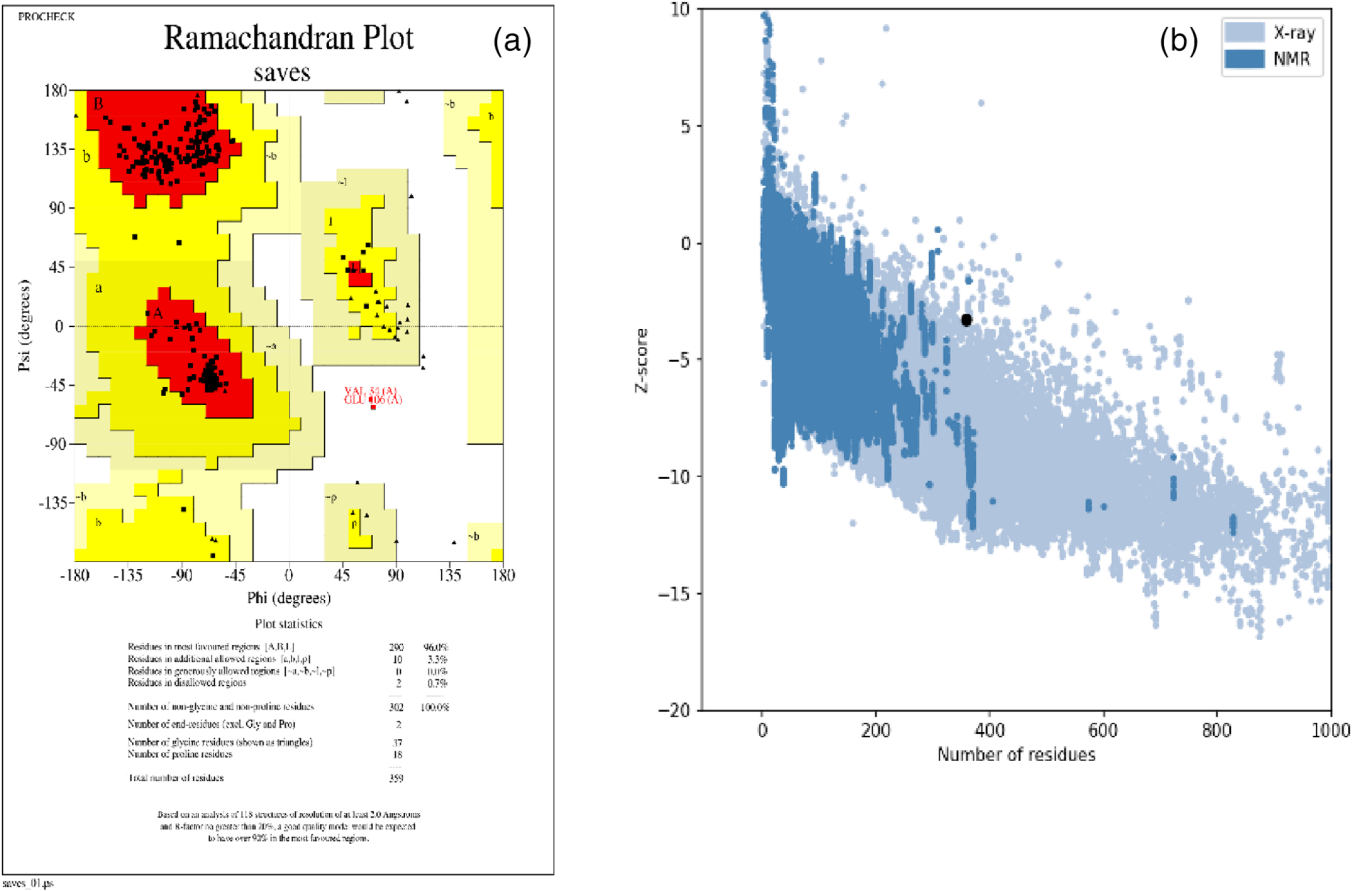
(a) Ramachandra plot before refinement, (b) *Z*‐score by the ProSA‐web‐server.

The loop refinement and energy minimization were carried out for obtaining the high quality of the predicted structure. The GalaxyRefine web‐server was used for the refinement of the initial ‘crude’ vaccine model that generated five model structures. Model 1 structure was the most significant one among all developed structures, based on several factors such as GDT‐HA (0.9777), RMSD (0.338) and MolProbity (1.384). The other parameters such as the clash value were 6.0, the low rotamers‐value was 0.0, and Rama favoured value was 97.8. This model 1 structure was also selected for additional study. trRosetta modelled 3D structure was subjected to the PROCHECK server, where the Ramachandran plot was generated and the output revealed 96% residues that were present in the favoured region (Figure 6a). The Ramachandran plot allows us to visualize energetically allowed and disallowed dihedral angles psi (*ψ*) and phi (*φ*). The *Z*‐score for the input vaccine (after refinement) was found to be −3.27 by the ProSA‐web‐server (Figure 6b). The overall results from RAMPAGE and ProSA‐web indicate the 3D modelled protein as of outstanding quality.

### 3.10 Prediction of discontinuous B‐cell epitopes

**FIGURE 7:**
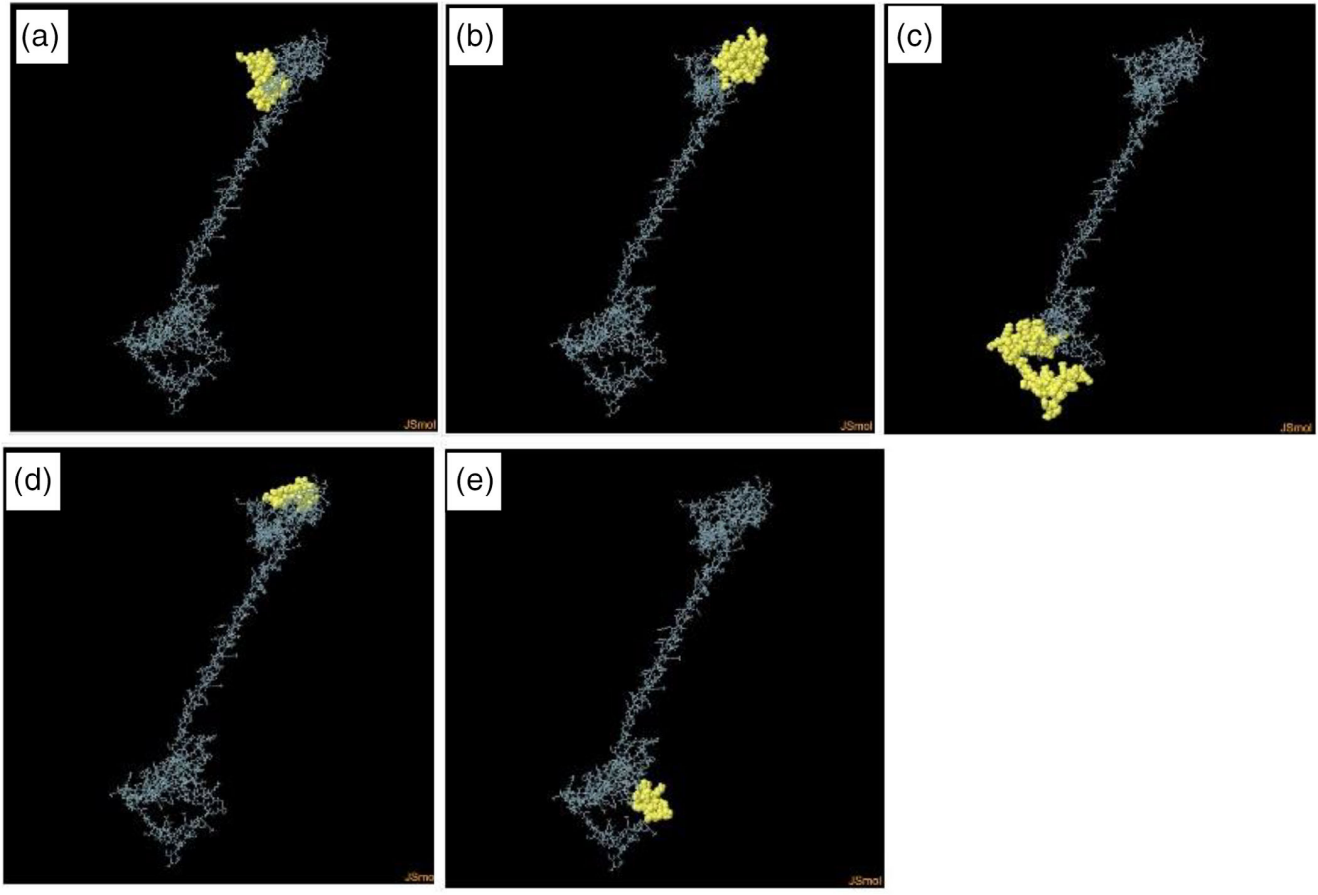
Three‐dimensional representation of conformational or discontinuous B cell epitopes of the designed multi‐epitope based vaccine. (a–e) A yellow surface represents the conformational or discontinuous B cell epitopes, and the bulk of the polyprotein is represented in grey sticks.

**FIGURE 8:**
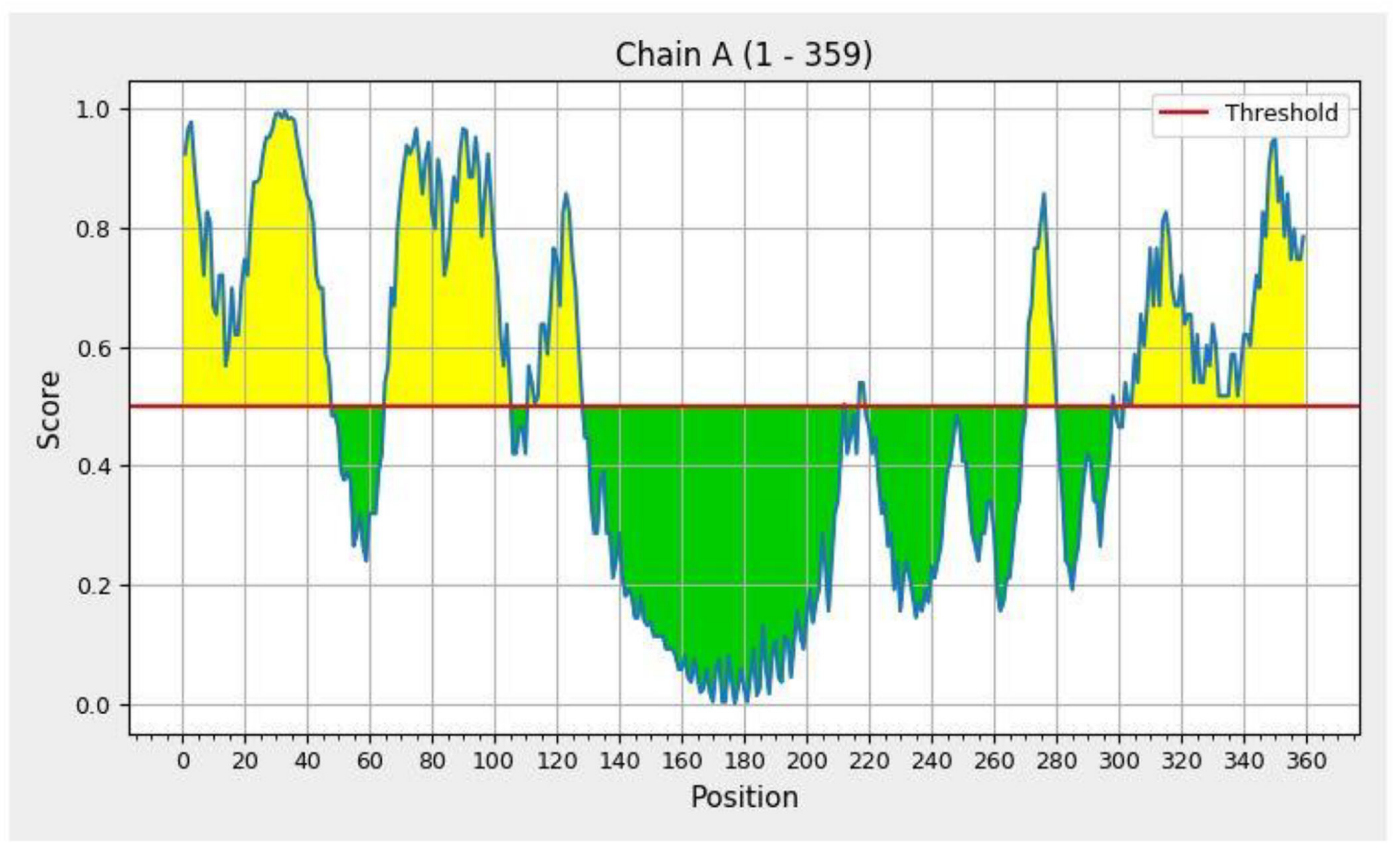
The individual score of discontinuous B cell epitopes was predicted in the multi‐epitope subunit vaccine.

One hundred eighty‐five residues were present in four discontinuous B‐cell epitopes, with values from 0.506 to 0.802. The size of the conformation epitopes ranged between 11 and 61 residues (Table 4). For the selection of the discontinuous peptides by Ellipro, the score value of the predicted peptides 0.66 or more was selected (Figure 7a–e and Table 5). Multiple discontinuous epitope residues were selected from vaccine sequence of varied epitope residues length such as 1–48 (48 epitope residues), between 65 and 106 (42 epitope residues), between 111 and 128 (18 epitope residues), between 270 and 281 (12 epitope residues) and between 301 and 359 (59 epitope residues). The score of each of the discontinuous epitopes has been shown in Figure 8.

**TABLE 4:** Predicted discontinuous epitope(s).

No.	Residues	No. of residues	Score
1	A:M1, A:A2, A:K3, A:L4, A:S5, A:T6, A:D7, A:E8, A:L9, A:L10, A:D11, A:A12, A:F13, A:K14, A:E15, A:M16, A:T17, A:L18, A:L19, A:E20, A:L21, A:S22, A:D23, A:F24, A:V25, A:K26, A:K27, A:F28, A:E29, A:E30, A:T31, A:F32, A:V34, A:T35, A:A36, A:A37, A:A38, A:P39, A:V40, A:A41, A:V42, A:A43, A:A44, A:A45, A:G46, A:A47, A:A48, A:P49	48	0.802
2	A:V65, A:I66, A:L67, A:E68, A:A69, A:A70, A:G71, A:D72, A:K73, A:K74, A:I75, A:G76, A:V77, A:I78, A:K79, A:V80, A:V81, A:R82, A:E83, A:I84, A:V85, A:S86, A:G87, A:L88, A:G89, A:L90, A:K91, A:E92, A:A93, A:K94, A:D95, A:L96, A:V97, A:D98, A:G99, A:A100, A:P101, A:K102, A:P103, A:L104, A:L105, A:V108, A:A109, A:E111, A:A112, A:A113, A:D114, A:E115, A:A116, A:K117, A:A118, A:K119, A:L120, A:E121, A:A122, A:A123, A:G124, A:A125, A:T126, A:V127, A:T128	61	0.762
3	A:P248, A:G249, A:I308, A:P309, A:N310, A:V311, A:S312, A:I313, A:K314, A:K315, A:G316, A:L317, A:V318, A:K319, A:K320, A:K321, A:N322, A:N323, A:I324, A:W325, A:V326, A:D327, A:V328, A:N329, A:S330, A:N331, A:S332, A:T333, A:V334, A:D335, A:M336, A:N337, A:K338, A:N339, A:T340, A:E341, A:L342, A:S343, A:N344, A:I345, A:K346, A:K347, A:S348, A:S349, A:K350, A:G351, A:D352, A:I353, A:H354, A:H355, A:H356, A:H357	52	0.677
4	A:G269, A:R270, A:Q271, A:F272, A:G273, A:P274, A:G275, A:P276, A:G277, A:L278, A:K279, A:V280, A:I281	13	0.644
5	A:N216, A:G217, A:P218, A:G219, A:P220, A:H222, A:L223, A:K303, A:N305, A:A306, A:K307	11	0.506

**TABLE 5:** Discontinuous peptides and predicted liner epitopes using Ellipro.

No.	Chain	Start	End	Peptide	No. of residues	Score
1	A	1	48	MAKLSTDELLDAFKEMTLLELSDFVKKFEETFEVTAAAPVAVAAAGAA	48	0.813
2	A	65	106	VILEAAGDKKIGVIKVVREIVSGLGLKEAKDLVDGAPKPLLE	42	0.81
3	A	301	359	KKKYNAKIPNVSIKKGLVKKKNNIWVDVNSNSTVDMNKNTELSNIKKSSKGDIHHHHHH	59	0.671
4	A	111	128	EAADEAKAKLEAAGATVT	18	0.662
5	A	270	281	RQFGPGPGLKVI	12	0.66

### 3.11 Disulfide engineering

**FIGURE 9:**
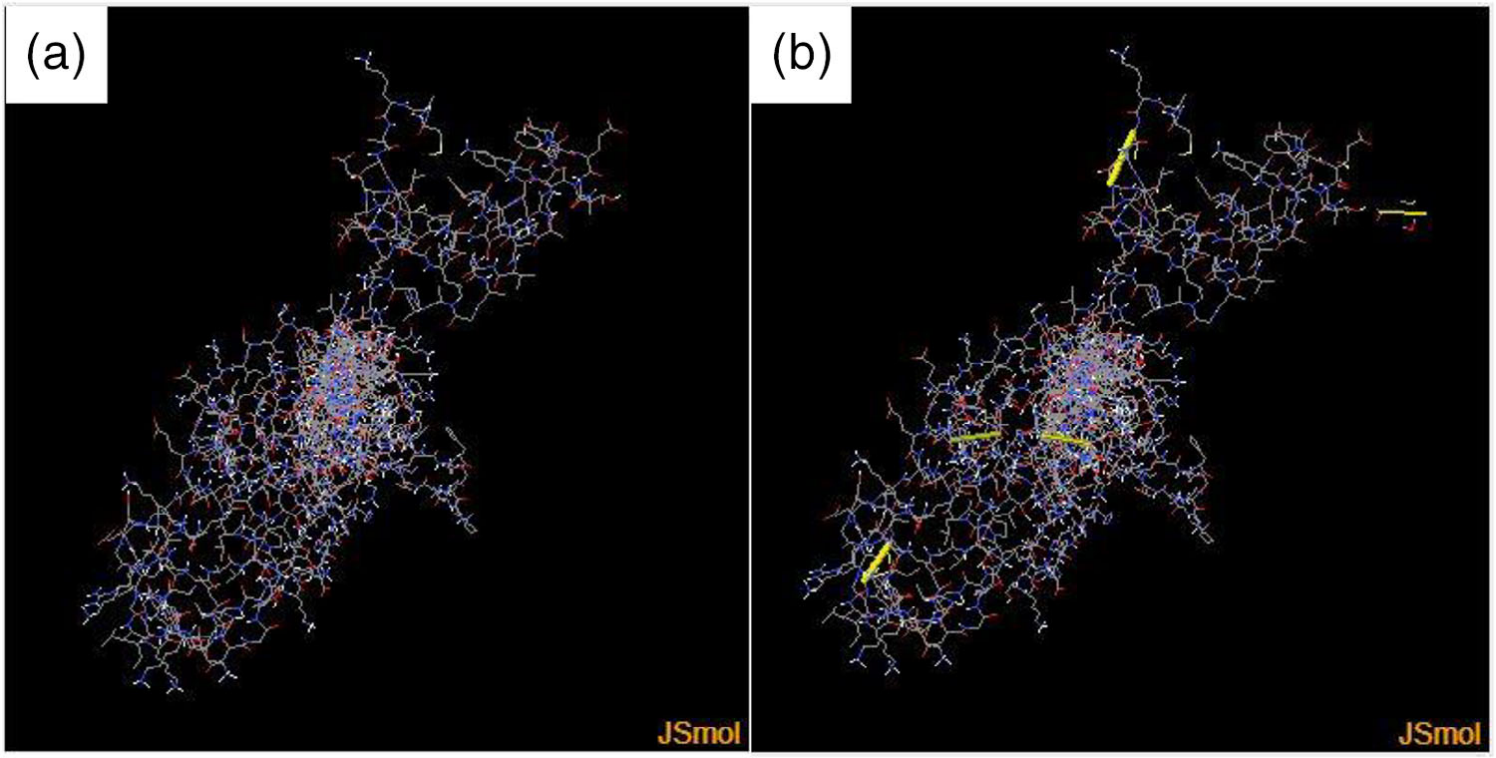
(a) Initial model without disulphide bonds. (b) Mutant model; the yellow stick represents the disulphide bond formation.

Disulfide engineering was performed to stabilize the vaccine construct by particular geometric confirmations (Hasan et al., 2019; Pandey et al., 2018). A total number of 27 pairs of amino acid residues were predicted to form a disulfide bond by the DbD2 server. Of which, only six residues, including LEU 4‐GLU 8, PHE 63‐ALA 113, GLY 76‐ALA 123, GLU 131‐ALA 134, PRO 248‐ASN 329 and ASP 335‐LYS 338, were replaced by cysteine residues, enabling disulfide bond formation following the residue assessment by chi3 and B‐factor energy parameters (Figure 9). Residue screening was performed on the basis of −87 to +97 chi3 values and <2.5 energy values.

### 3.12 Molecular docking of the final vaccine with immune receptor

**FIGURE 10:**
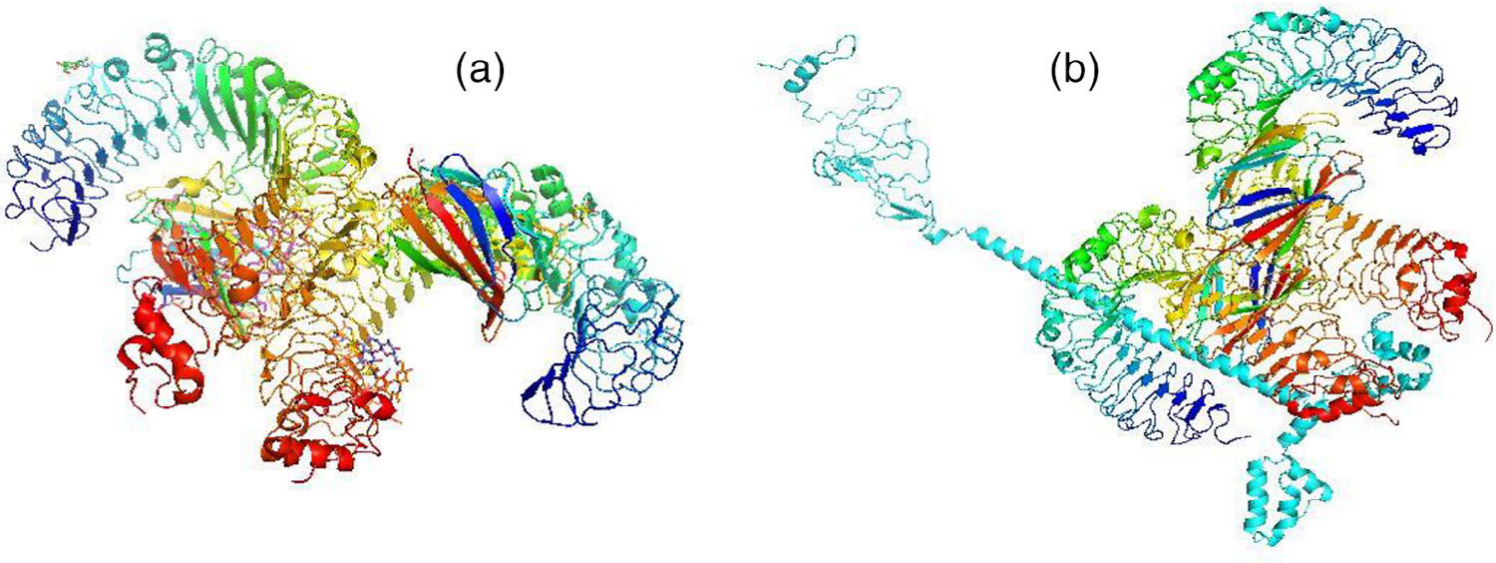
(a) Three‐dimensional (3D) structure of Toll‐like receptor 4 (TLR4), (b) docked product of TLR4 and vaccine construct by Cluspro.

The interaction between the immune cell and the vaccine construct is required for an effective and stable immune response. The Cluspro program yielded 10 different clusters, which had higher interaction energies. Based on the energy score, the first cluster was selected that had better energy than other clusters (Figure 10).

### 3.13 Molecular dynamics simulation

**FIGURE 11:**
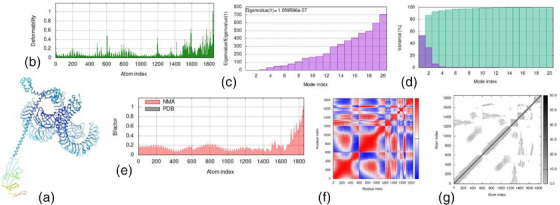
The of molecular dynamics simulation results: (a) normal mode analysis (NMA) mobility, (b) deformability, (c) eigenvalues, (d) variance, (e) B‐factor, (f) co‐variance map (correlated (red), uncorrelated (white) or anti‐correlated (blue) motions) and (g) elastic network (darker grey regions indicate stiffer regions).

The results of molecular dynamics simulation and normal mode analysis (NMA) of the vaccine construct and TLR4 docked complex are illustrated in Figure 11a. The simulation study was conducted to determine the movement of molecules and atoms in the vaccine construct. The deformability graph of the complex shows the peaks in the graphs, which represent the regions of the protein with deformability (Figure 11b). The eigenvalue of the complex is 1.059586e − 07 as shown in Figure 11c. The variance graph displays the cumulative variance as indicated by green colour and individual variance by purple colour (Figure 11d). The B‐factor graph gives a clear visualization of the relation of the docked complex between the NMA and the PDB sector (Figure 11e). The covariance map of the complex indicates the correlated motion between a pair of residues, which is indicated by red colour, uncorrelated motion indicated by white colour and anti‐correlated motion by blue colour (Figure 11f). The complex's elastic map shows the relation between the atoms and darker grey regions, indicating stiffer regions (Figure 11g).

### 3.14 Codon optimization and in silico cloning

**FIGURE 12:**
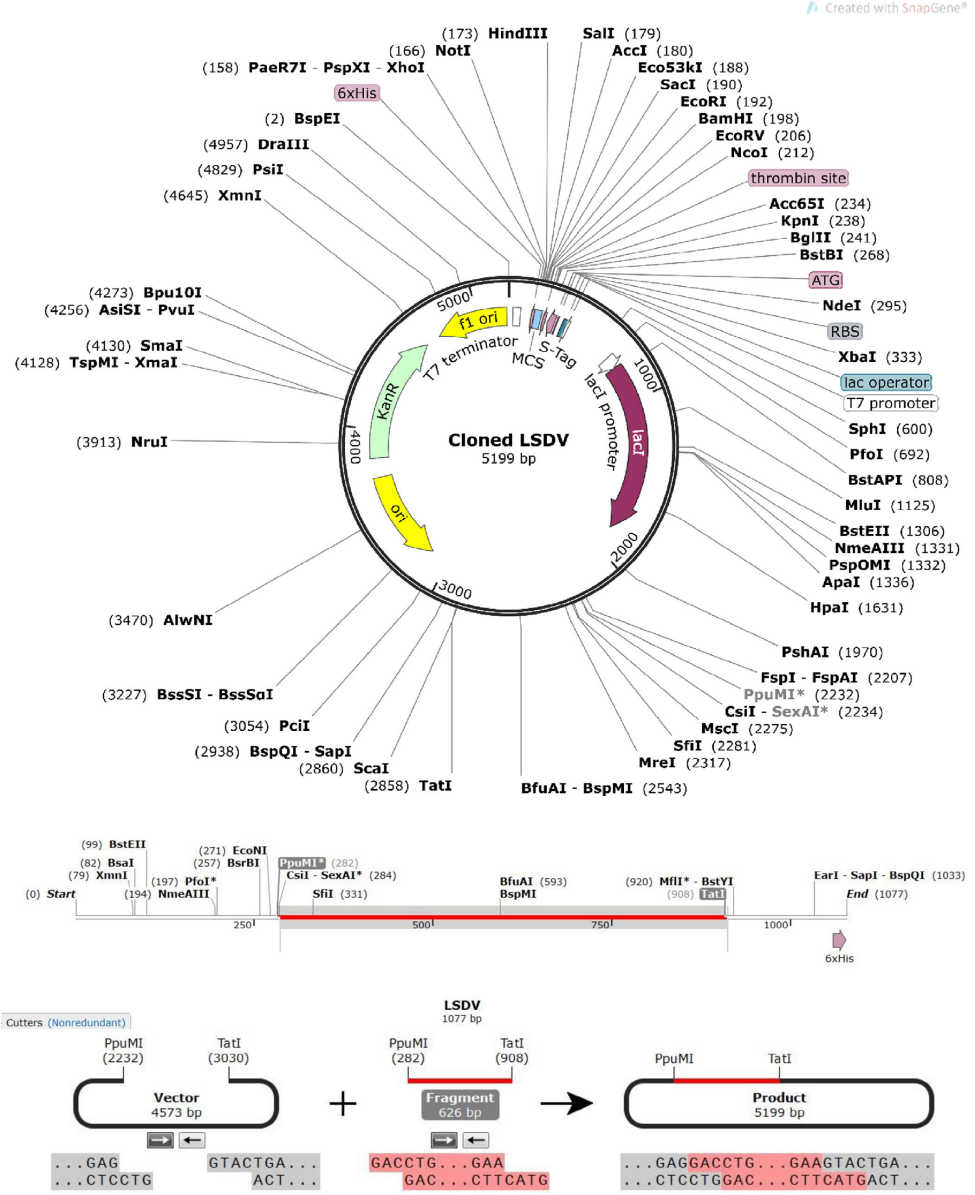
Expression vector pET29a (+). In silico restriction cloning of the multi‐epitope vaccine sequence into the pET29a (+) expression vector using SnapGene software free‐trial (https://www.snapgene.com/free‐trial/).

JCat was used for maximal protein expression in *E. coli* (strain K12) and *D. melanogaster*. The optimized codon sequence contained 1077 nucleotides. Initial CAI value of pasted sequence was 0.3383, and GC content was 66.48%. After adaptation, improved CAI value for *E. coli* (strain k12) was 0.4857, GC content was 66.48%, and GC content of *E. coli* was 50.73%. For *D. melanogaster*, CAI of pasted sequence was 0.8879, and GC content was 66.4809%. After adaptation improved CAI value was 0.9308, GC content 66.945% and GC content of *D. melanogaster* was 50.73%. Based on accuracy, the expression system of TLR4 and higher CAI value *D. melanogaster* was chosen as host. To perform in silico cloning, the multi‐epitope vaccine construct was examined for restriction enzyme sites, including the PpuMI and TatI restriction sites, and was used for in silico cloning in the pET29a (+) vector. A construct of 5199 bp was achieved after the introduction of the vaccine sequence into the pET29a (+) vector using SnapGene software (Figure 12).

### 3.15 Immune simulation

**FIGURE 13:**
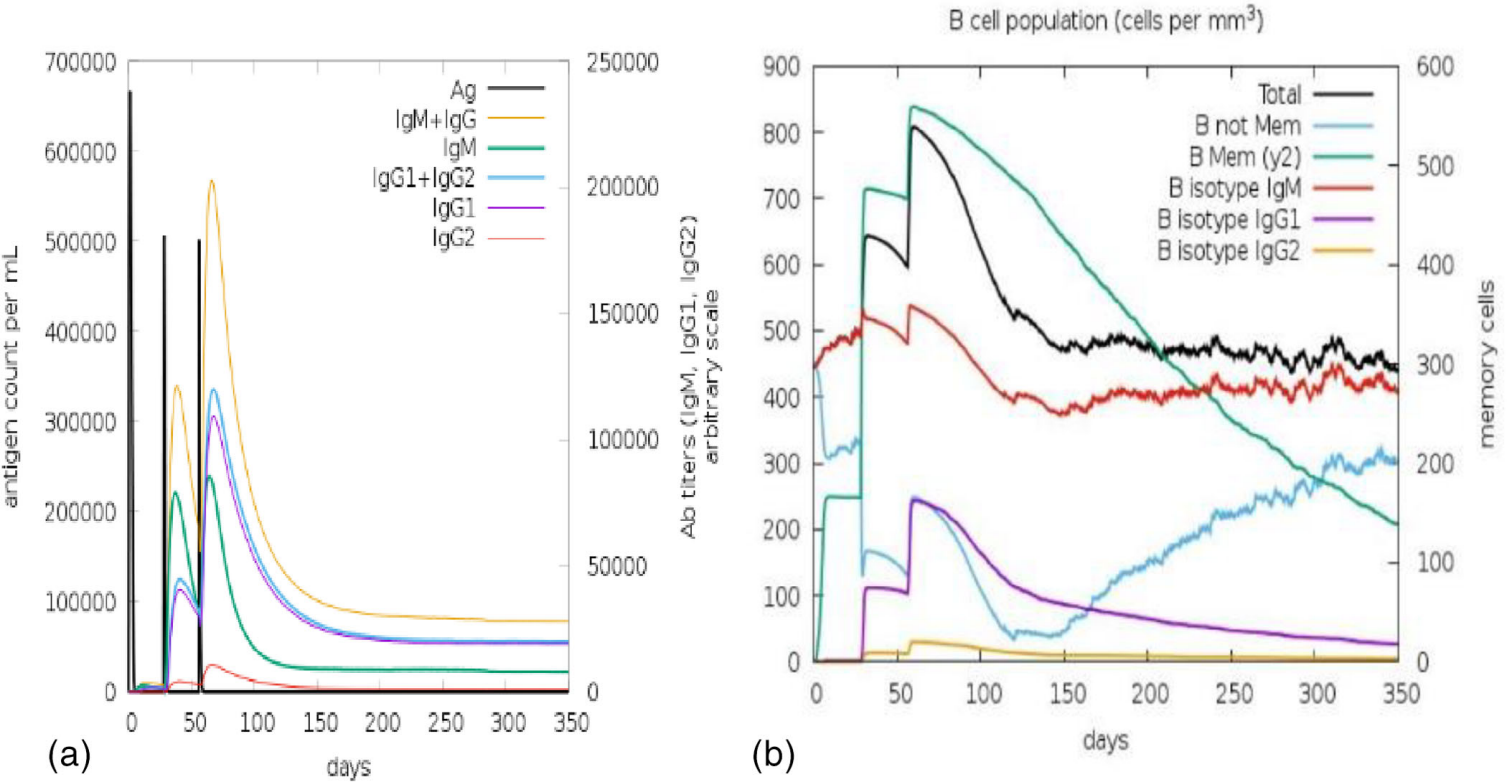
In silico immune simulation of vaccine construct: (a) immunoglobulin production in response to antigen injections (black vertical lines); specific subclasses are shown as coloured peaks, and (b) the evolution of B‐cell populations after the three injections.

**FIGURE 14:**
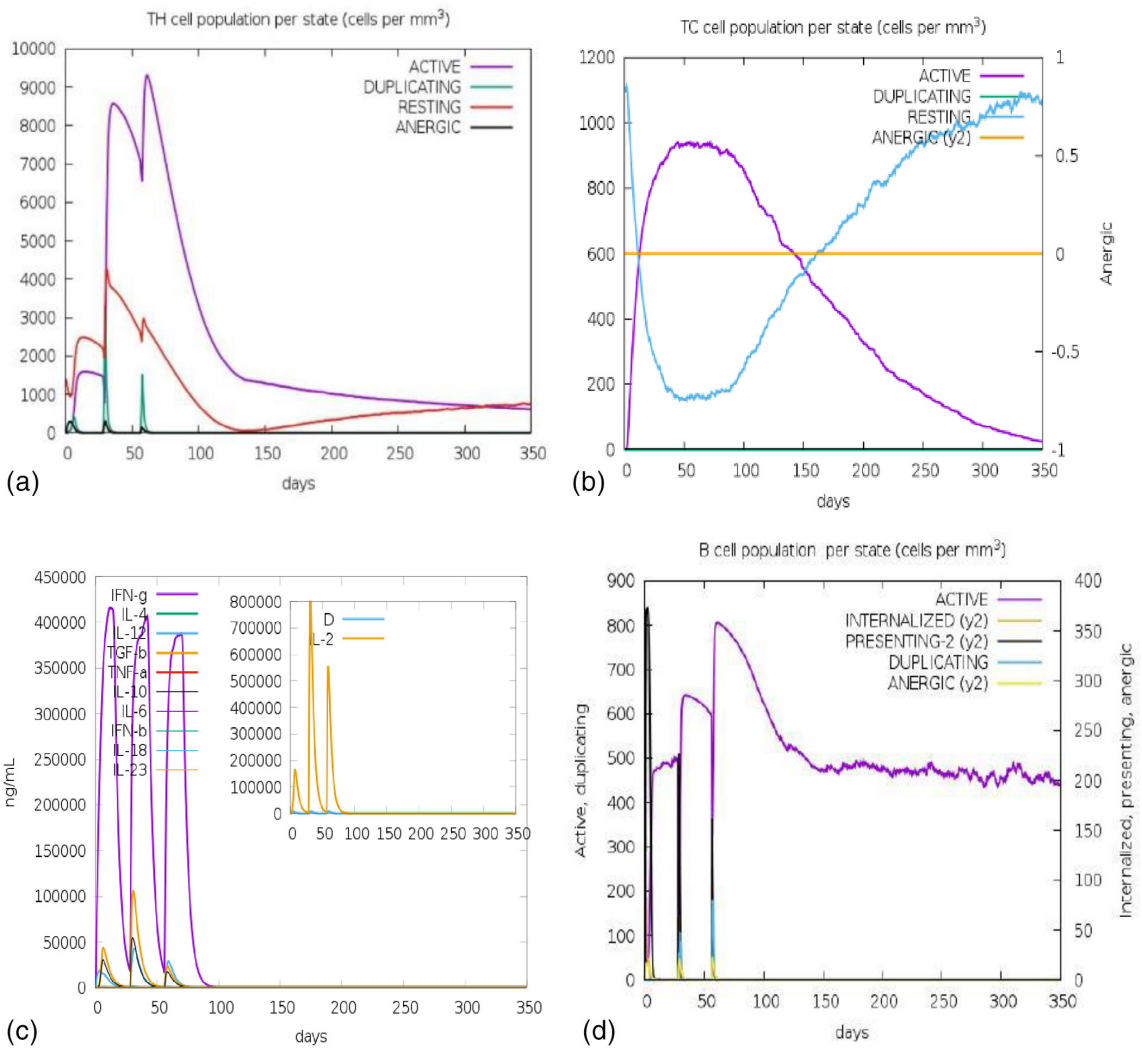
In silico immune simulation of vaccine construct. (a) The evolution of T‐helper and (b) cytotoxic cell populations per state after the injections. The resting state represents cells not presented with the antigen, whereas the anergic state characterizes tolerance of the T‐cells to the antigen due to repeated exposures. (c) The main plot shows cytokine levels after the injections. The insert plot shows IL‐2 level with the Simpson index, D shown by the dotted line. D is a measure of diversity. Increase in D over time indicates emergence of different epitope‐specific dominant clones of T‐cells. The smaller the D value, the lower the diversity. (d) Active B cell population state after vaccination.

The immune simulation of the vaccine construct was performed with C ImmSim server, which studies the successive and effective immune responses of the state of the cell and the memory of immune cells by a mechanism that increases their half‐life. ImmSim server immune simulation outcomes confirmed consistency with real immune reactions, as illustrated by high IgM levels in primary response. Moreover, there was an increase in the B‐cell population, characterized by an increase in the immunoglobulin (IgG1 + IgG2, IgM and IgG + IgM) expression, resulting in a decrease in antigen concentration (Figure 13a,b). There was a clear increase in the population of helper and cytotoxic T‐cells with increased memory (Figure 14a,b). An increased IFN‐γ production and active B‐cell population were identified after immunization (Figure 14c,d).

## 4 DISCUSSION

Vaccines are important tools for the host defence against particular pathogen. The conventional vaccine development process is extremely meticulous, expensive and time‐consuming as the identification of immunodominant protein of a pathogen is tedious process. With the advent of immunoinformatics tools, the design and development of a vaccine have become fast and successful (Shilpa & Shivakumar, 2022). Immunoinformatics tools help in detecting the immunogenic proteins with diverse immune‐dominant epitopes, which can stimulate both humoral and cell‐mediated immune responses against the pathogen (Mugunthan & Mani Chandra, 2021). Therefore, a multi‐epitope‐based peptide vaccine can be easily designed with immunogenic proteins of a pathogen. Recently, a large number of peptide vaccines are under development, majority of which are for human infectious diseases and tumours (Kar et al., 2020; Kolesanova et al., 2013, 2019; Rapin et al., 2010). However, a limited number of studies are reported in the field of in silico vaccine development for poultry and animals. Multi‐epitope vaccine development for animal diseases such as foot and mouth disease (Liu et al., 2017) and animal trypanosomiasis (Michel‐Todó et al., 2020) have been reported, which offered effective immunity as evaluated with currently available vaccines (Mugunthan & Harish, 2021). Several studies utilized the immunoinformatics tools to design multi‐epitope vaccines against infectious poultry diseases (Hasan et al., 2019; Ingale & Goto, 2014; Osman et al., 2016; Unni et al., 2019).

By the use of immunoinformatics tools, evaluation of complete antigenic epitopes and molecular modelling for the probable binding with host proteins are possible (Jabbar et al., 2018; Tosta et al., 2021). The in silico validations like molecular docking and in silico cloning and immune simulation were included in this study to design and develop multi‐epitope vaccine construct against LSDV infection (Ashfaq et al., 2021; Behbahani et al., 2021; Hossain et al., 2021; Sanches et al., 2021). The purpose of multi‐epitope vaccine is to enhance the humoral and cell‐mediated immune response by identifying the specific MHC I, MHC II and B‐cell epitopes from the antigenic proteins. The selected proteins were found antigenic, non‐allergic and non‐toxic during initial analyses of the vaccine construct. A good vaccine candidate should initiate proper immune response without an allergic reaction, and expectedly, our vaccine construct was antigenic but non‐allergic in nature. Vaccine response can be enhanced by incorporating adjuvant in the vaccine construct (Shey et al., 2019); therefore, 50S ribosomal protein L7/L12 (UniProt ID: P9WHE3) was used as an adjuvant at the N‐terminal followed by a sequence of various MHC I, MHC II and B‐cell epitopes present in the peptide vaccine (Henderson & Jensen, 2006). In this study, the MW of the vaccine construct and the theoretical pI signifies the basic nature of the vaccine construct. The aliphatic index and low GRAVY score indicate the thermostable and hydrophilic nature of the vaccine candidate (Abdi et al., 2022; Susithra Priyadarshni et al., 2022; Validi et al., 2018). Moreover, the GRAVY value 0.008 of the vaccine suggesting that the vaccine may interact with water (Abdi et al., 2022), and this vaccine is ideal for its usage in endemic areas (Bachmann & Jennings, 2010; Omoniyi et al., 2022). The secondary structure analysis of the vaccine construct indicated that the alpha‐helices dominating the structure followed by random coil and beta sheets, which are suggestive of the existence of natively unfolded protein regions that can be identified by antibodies, produced in response to infection (Bibi et al., 2021). The tertiary structure of the construct was selected based on the highest TM score, and our construct had a lower RMSD score of 0.338, indicating better stability (Yang et al., 2021). The structure was further refined, and a better structure was obtained after refinement. According to Ramachandran plot, 96% residues were present in the favoured region, which is higher than the acceptable range (Omoniyi et al., 2022; Yang et al., 2021). The *Z*‐score determined by ProSA indicates overall model quality, in which the negative values signify that no components of the structural model are flawed (Shantier et al., 2022). The estimated *Z*‐score for the designed vaccine candidate was −3.27, indicating that it is satisfactory. The predicted discontinuous and continuous B‐cell epitopes of the vaccine construct showed possible interaction with antibodies and were flexible. Furthermore, disulfide engineering was performed to stabilize the vaccine construct. The interaction prototype of the vaccine construct with TLR4 was analysed by molecular docking, which indicated a better interaction and the docked complex was energetically viable. Expression systems play a crucial role in the serological immunoreactivity screening process that is also critical for vaccine validation. Codon optimization was carried out in *D. melanogaster* to ensure that the vaccination protein was fully expressed (Akmammedov et al., 2017; Gori et al., 2013). Codon optimization was done for the *D. melanogaster* strain; the estimated GC content and CAI indicated enhanced transcriptional and translational efficiency (Omoniyi et al., 2022). A *D. melanogaster*‐based expression system selected because it enables recombinant protein production on a large scale at a low cost (Omoniyi et al., 2022). The expression of codon was adequate, and the administration of the multi‐epitope vaccine induced a strong immune response. The immune response to the chimeric vaccine was comparable with actual immune responses with higher tertiary and secondary responses. Current commercial vaccines are widely employed in commercial farms; nevertheless, these vaccines cannot aid in control during the abrupt onset of LSD infection; rigorous biosecurity must be observed to manage and eradicate the infection. The current study addresses the newly formulated multi‐epitope vaccine candidate using robust immunoinformatics tools, and the results show that the designed vaccine construct has every aspect of being developed as an effective vaccine candidate, such as immune specificity, small size and the absence of adverse effects.

## 5 CONCLUSION

This study uses immunoinformatic tools to design and develop a multi‐epitope vaccine against LSDV. The candidate vaccine was found highly immunogenic, non‐allergenic, non‐toxic and antigenic with a high affinity for the TLR4 immune receptor. Furthermore, the simulated immune response showed cellular and humoral immune responses, as well as efficient memory cell development. Although this study should provide a platform for new vaccine development, however, these in silico findings should be further validated by in vitro and in vivo studies before further advancement.

## AUTHOR CONTRIBUTIONS

*Concept and investigation*: Md. Salauddin. *Data arrangement and analysis*: Md. Salauddin. *Initial draft*: Md. Salauddin, Mohammad Enamul Hoque Kayesh and Md. Suruj Ahammed. *Critical review and writing the final version of the manuscript*: Md. Salauddin, Mohammad Enamul Hoque Kayesh, Sukumar Saha and Md. Golzar Hossain. All authors approved the final version of the manuscript.

## CONFLICT OF INTEREST STATEMENT

The authors declare no conflicts of interest.

## FUNDING INFORMATION

The authors would like to express their gratitude to City Bank for providing a research grant (Project No.: 2023/13/Other) through Bangladesh Agricultural University.

### ETHICS STATEMENT

This is a bioinformatics based study. Ethical approval is not required.

### PEER REVIEW

The peer review history for this article is available at https://publons.com/publon/10.1002/vms3.1438.

## Data Availability

All the data are available in the manuscript.
